# Acute exacerbation of chronic obstructive pulmonary disease in United States emergency departments, 2010–2018

**DOI:** 10.1186/s12890-023-02518-0

**Published:** 2023-06-20

**Authors:** Chiat Qiao Liew, Shu-Hsien Hsu, Chia-Hsin Ko, Eric H. Chou, Jeffrey Herrala, Tsung-Chien Lu, Chih-Hung Wang, Chien-Hua Huang, Chu-Lin Tsai

**Affiliations:** 1 Department of Emergency Medicine, National Taiwan University Hospital, 7 Zhongshan S. Rd, Taipei, 100 Taiwan; 2 Department of Emergency Medicine, College of Medicine, National Taiwan University, Taipei, Taiwan; 3 Department of Emergency Medicine, Baylor Scott and White All Saints Medical Center, Fort Worth, TX USA; 4 Department of Emergency Medicine, Highland Hospital-Alameda Health System, Oakland, USA

**Keywords:** Chronic obstructive pulmonary disease, Emergency department, Epidemiology, Exacerbation

## Abstract

**Objectives:**

Little is known about the recent status of acute exacerbation of chronic obstructive pulmonary disease (AECOPD) in the U.S. emergency department (ED). This study aimed to describe the disease burden (visit and hospitalization rate) of AECOPD in the ED and to investigate factors associated with the disease burden of AECOPD.

**Methods:**

Data were obtained from the National Hospital Ambulatory Medical Care Survey (NHAMCS), 2010–2018. Adult ED visits (aged 40 years or above) with AECOPD were identified using International Classification of Diseases codes. Analysis used descriptive statistics and multivariable logistic regression accounting for NHAMCS’s complex survey design.

**Results:**

There were 1,366 adult AECOPD ED visits in the unweighted sample. Over the 9-year study period, there were an estimated 7,508,000 ED visits for AECOPD, and the proportion of AECOPD visits in the entire ED population remained stable at approximately 14 per 1,000 visits. The mean age of these AECOPD visits was 66 years, and 42% were men. Medicare or Medicaid insurance, presentation in non-summer seasons, the Midwest and South regions (vs. Northeast), and arrival by ambulance were independently associated with a higher visit rate of AECOPD, whereas non-Hispanic black or Hispanic race/ethnicity (vs. non-Hispanic white) was associated with a lower visit rate of AECOPD. The proportion of AECOPD visits that were hospitalized decreased from 51% to 2010 to 31% in 2018 (p = 0.002). Arrival by ambulance was independently associated with a higher hospitalization rate, whereas the South and West regions (vs. Northeast) were independently associated with a lower hospitalization rate. The use of antibiotics appeared to be stable over time, but the use of systemic corticosteroids appeared to increase with near statistical significance (p = 0.07).

**Conclusions:**

The number of ED visits for AECOPD remained high; however, hospitalizations for AECOPD appeared to decrease over time. Some patients were disproportionately affected by AECOPD, and certain patient and ED factors were associated with hospitalizations. The reasons for decreased ED admissions for AECOPD deserve further investigation.

## Introduction

Chronic obstructive pulmonary disease (COPD) remains a leading cause of mortality and morbidity in the U.S. and worldwide [1]. Acute exacerbations of COPD (AECOPD) are important adverse events in the natural history of the disease [2, 3]. Frequent exacerbations (2 + per year) are associated with higher mortality, reduced quality of life and a faster lung function decline than those with infrequent exacerbations (0–1 per year) [2, 3]. AECOPD often results in emergency department (ED) visits, accounting for approximately 0.6–1.5 million ED visits each year in the U.S [4, 5]. Understanding the epidemiology of ED visits for AECOPD may help monitor the disease burden and quality of care for stable COPD [6–8].

Some earlier research has reported the epidemiology of ED visits for AECOPD. For example, Tsai et al. found a persistently high burden of ED visits for AECOPD during 1993–2005 [6]. In addition, the overall concordance with guideline-recommended care for AECOPD was only moderate during that study period [6]. Afterward, there have been few studies investigating ED visits for AECOPD patients [4, 9, 10]. No studies, however, have examined this issue since the inception of the Hospital Readmissions Reduction Program (HRRP) in 2014 [11], which may have affected the rate of hospitalization through the ED. In addition, several COPD practice guidelines, including the Global Initiative for Chronic Obstructive Lung Disease (GOLD) guidelines [12] and American Thoracic Society (ATS) guidelines [13], highlight important advances in the management of stable COPD. Specifically, whether new therapeutics, such as dual bronchodilators and/or long-term non-invasive ventilation use, would have affected ED visits and hospitalizations for AECOPD remains unknown.

To address these knowledge gaps, we analyzed recent data from the National Hospital Ambulatory Medical Care Survey (NHAMCS) from 2010 to 2018. The objectives of this study were (1) to describe the incidence of and hospitalization for AECOPD in the ED, and (2) to investigate factors associated with the disease burden of AECOPD.

## Methods

### Study design and setting

The NHAMCS is a cross-sectional, multistage probability sample of visits to non-institutional general and short-stay hospitals, excluding federal, military, and Veterans Administration hospitals, located in the 50 states and the District of Columbia. The NHAMCS is conducted annually by the National Center for Health Statistics (NCHS). It covers geographic primary sampling units, hospitals within primary sampling units, EDs within hospitals, and patients within EDs. The number of EDs sampled is approximately 300–400 per year. Trained ED staff collected clinical information during a randomly assigned four-week period for each of the sampled EDs using a structured Patient Record Form (PRF). Data included patient demographics, reasons for the visit, diagnoses, procedures, medications given at the visit, and the basic characteristics of the treating physician and hospital. Quality control was performed using a two-way independent verification procedure with a 10% sample of the records. The non-response rate for most items was < 5%. The coding error rates were < 2% [14]. Because the NHAMCS contains publicly available, de-identified data, the National Taiwan University Hospital Institutional Review Board exempted this study from review.

### Study population

NHAMCS data from 2010 to 2018 were used in this analysis. We included ED visits for patients 40 years of age or older for this analysis because individuals younger than 40 years are less likely to have COPD. This also decreased the likelihood of including visits for asthma that were misclassified as COPD. For this study, adults were defined as persons aged 40 or older throughout the text for brevity and consistency. Up to five diagnosis fields in the NHAMCS were coded according to the International Classification of Diseases, Ninth Revision, Clinical Modification (ICD-9-CM), or ICD-10. For the current analysis, we used ICD-9-CM codes (491 [chronic bronchitis], 492 [emphysema], or 496 [COPD not otherwise specified]), and ICD-10 codes (J41-J44) to define COPD. These codes have been validated in administrative data [15]. We identified patient visits in which any of the three COPD codes were listed in the primary ED diagnosis field as ED visits for AECOPD.

### Variables

To preserve consistency across years, race/ethnicity was recoded as non-Hispanic white, non-Hispanic black, Hispanic, and other. Insurance was recoded as private, Medicare, Medicaid or other state-based programs, self-pay, and other. The US regions represented standardized geographical divisions, as defined by the US Census Bureau (Northeast, Midwest, South, and West) [16]. Up to five reasons for each ED visit were coded using the Reason for Visit Classification for Ambulatory Care, a standardized sourcebook used in NCHS studies. Chronic comorbid conditions were ascertained based on the PRF, including diabetes mellitus, hypertension, coronary artery disease, and cancer. Data on disease severity/urgency included triage levels, vital signs at triage, and pain scores. A number of procedures were documented on the PRF, including endotracheal intubation. Imaging performed in the ED was also recorded, including computed tomography (CT) scan. Up to eight medications were recorded during an ED visit. The therapeutic category of medication was based on the Multum Lexicon Drug Database [17]. We identified the following AECOPD medications using the Multum codes: (1) systemic corticosteroids (code 301), and (2) antibiotics (codes 012 [miscellaneous antibiotics], 014–016 [quinolones, sulfonamides, tetracyclines], 159–162 [first to fourth generation cephalosporins], 222–226 [penicillins and penicillins/beta-lactamase inhibitors], 304–305 [macrolides and ketolides], 379 [fifth generation cephalosporins], 492 [cephalosporins/beta-lactamase inhibitors]), and (3) methylxanthine (code 126). Visit disposition was recorded for each ED visit. For ED visits resulting in hospitalizations, inpatient mortality, and hospital length of stay (LOS) were recorded.

### Outcome measures

The primary outcome measure was the AECOPD incidence (visit) rate in the ED. We calculated this as the number of ED visits for AECOPD divided by the total number of adult ED visits. The co-primary outcome measure was the admission rate for ED AECOPD visits.

### Statistical analysis

Stata 16.0 (StataCorp, College Station, TX) was used to adjust the variances for the NHAMCS estimates, accounting for the complex design of the survey. Statistics from the NHAMCS were derived by a multistage estimation procedure, including (1) inflation by reciprocals of the sampling selection probabilities; (2) adjustment for nonresponse; and (3) a population weighting ratio adjustment. Standard errors (SEs) were calculated for the NHAMCS estimates. All statistical tests were based on reliable estimates that had at least 30 cases and a < 30% relative SE in the sample data, according to the NCHS recommendations. Descriptive data were presented as proportions (with 95% confidence intervals [CIs]), means (with SEs), or medians (with interquartile ranges [IQRs]). We used the weighted chi-square test to analyze the differences between proportions. Logistic regression models were used to test for significant changes in the primary outcomes (ED visit and admission rates for AECOPD) during the study period, in which the calendar year was a linear independent variable. As a sensitivity analysis, we repeated the trend analyses using the more restrictive exacerbation codes (ICD-9-CM code 491.21 and ICD-10 code J44.1). Regression discontinuity analysis was used to test the change in slope and intercept of hospitalization rates before and after 2014, when the HRRP was implemented. Multivariable logistic regression modeling was performed to evaluate independent factors associated with the two primary outcomes. The factors included in the model were age, sex, race/ethnicity, insurance, season, weekend, time of presentation, geographic region, and arrival mode. Odds ratios (ORs) are presented with 95% CI. All P values are two-sided, with a P value < 0.05 considered statistically significant.

## Results

**Fig. 1:**
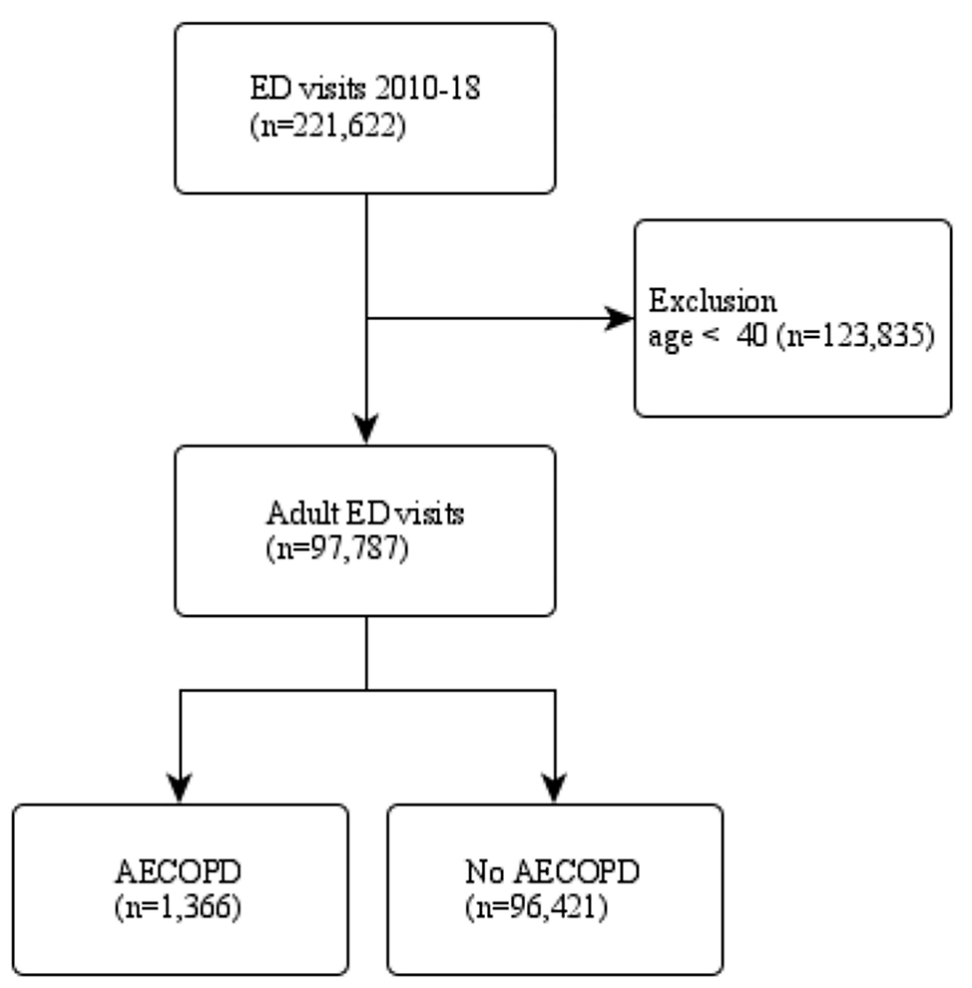
The patient selection process

From 2010 to 2018, 221,622 ED visits were recorded in the NHAMCS. After excluding 123,835 visits from patients aged less than 40 years, a total of 97,787 adult ED visits were included in the analysis. Of these adult ED visits, 1,366 visits were labeled as AECOPD, and 96,421 were non-AECOPD. The flowchart is presented in Fig. 1.

### Characteristics of ED visits for AECOPD

After weighting, there were an estimated 7,508,000 adult ED visits for AECOPD from 2010 to 2018 in the United States. The clinical characteristics of these ED visits are summarized in Table 1. The elderly population (age 65+) accounted for 51% of the ED AECOPD population. The proportion of visits was higher in women (58.2%), in non-Hispanic whites (76.4%), and in patients with Medicare insurance (59.8%). The visits occurred more often in the winter months and presented more often in the day shift (48%). The proportion of visits was higher in the South (42.8%). Approximately 38% of patients required ambulance services. The mean number of comorbidities was 3. The most common reasons for the visits were dyspnea and cough. Most patients were triaged at level 3. The mean triage vital signs showed mild tachycardia, tachypnea, and hypoxia. Very few (0.7%) of the ED visits resulted in intubation. Approximately 81% received chest radiography examination, whereas the use of CT scan was relatively low (9% for chest CT and 12% for any CT). Systemic corticosteroids and bronchodilators were commonly administered in AECOPD visits, whereas antibiotics were administered in 40% of the visits. The median length of ED stay was about 3.5 h. 37% of the visits were hospitalized. Among those who were hospitalized, the median length of hospital stay was 4 days, and 1% of the patients died during the hospital stay.

**Table 1:** Baseline clinical characteristics of emergency department patients with acute exacerbation of chronic obstructive pulmonary disease, 2010–2018

Variable	Unweighted Number n (%), mean (SD) or median (IQR)	Weighted Number or Weighted Mean/Median	Weighted percentage (95% CI) or IQR
Total	1,366	7,508,000	
Age group			
40–64	646 (47.3)	3,672,000	48.9 (45.2–52.6)
65+	720 (52.7)	3,836,000	51.1 (47.4–54.8)
Sex			
Male	588 (43.1)	3,137,000	41.8 (38.1–45.5)
Female	778 (57.0)	4,371,000	58.2 (54.5–61.9)
Race/ethnicity			
Non-Hispanic White	1,045 (76.5)	5,733,000	76.4 (72.1–80.1)
Non-Hispanic Black	219 (16.0)	1,252,000	16.7 (13.5–20.5)
Hispanic	67 (4.9)	347,000	4.6 (3.2–6.6)
Other	35 (2.6)	176,000	2.3 (1.1–4.7)
Insurance			
Private insurance	187 (14.4)	996,844	14.0 (11.1–17.4)
Medicare	785 (60.3)	4,260,000	59.8 (55.7–63.7)
Medicaid or other state-based program	221 (17.0)	1,281,000	18.0 (15.2–21.2)
Self-pay (uninsured)	81 (6.2)	442,000	6.2 (4.2–3.2)
Other	29 (2.2)	145,000	2.0 (1.3–3.2)
Season			
Spring (Mar. – May)	366 (26.8)	1,852,000	24.7 (20.9–28.8)
Summer (Jun. – Aug.)	272 (19.9)	1,466,000	19.5 (16.5–22.9)
Fall (Sep. – Nov.)	346 (25.3)	1,936,000	25.8 (22.5–29.4)
Winter (Dec. – Feb.)	382 (28.0)	2,254,000	30.0 (26.5–33.8)
Weekend	374 (27.4)	2,105,000	28.0 (25.2–31.1)
Time of presentation			
7:00 am to 2:59 pm	627 (46.6)	3,561,000	48.0 (43.9–52.0)
3:00 pm to 10:59 pm	488 (36.3)	2,684,000	36.2 (32.7–39.8)
11:00 pm to 6:59 am	231 (17.2)	1,177,000	15.9 (13.5–18.6)
Geographic region			
Northeast	244 (17.9)	1,035,000	13.8 (10.9–17.3)
Midwest	433 (31.7)	2,213,000	29.5 (23.3–36.5)
South	496 (36.3)	3,216,000	42.8 (36.3–49.6)
West	193 (14.1)	1,044,000	13.9 (10.1–18.8)
Arrival by ambulance	490 (37.0)	2,773,000	37.9 (33.9–42.1)
Number of comorbid conditions, mean	2.8 (1.9)	3.0	2.8–3.2
Most common chief complaints			
Dyspnea	1,051 (76.9)	5,859,000	78.0 (74.2–81.5)
Cough	354 (25.9)	2,131,000	28.4 (24.3–32.9)
Triage level			
1	16 (1.5)	57,000	1.0 (0.4–2.4)
2	290 (6.3)	1,631,000	27.3 (23.1–31.9)
3	633 (57.4)	3,382,000	54.9 (51.3–58.5)
4	147 (13.3)	933,000	15.6 (11.9–20.3)
5	17 (1.5)	72,000	1.2 (0.6–2.3)
Triage vital signs			
Body temperature, mean, °C	36.7 (0.5)	36.8	36.7–36.8
Heart rate, mean, beats per min	93.3 (31.4)	91.8	90.4–93.2
Respiratory rate, mean, breaths per min	22.2 (6.5)	22.0	21.6–22.5
Oxygen saturation, mean, %	94.0 (6.3)	94.2	93.7–94.7
Systolic blood pressure, mean, mmHg	140.6 (25.2)	140.8	139.1-142.4
ED management			
Intubation	9 (0.7)	50,000	0.7 (0.3–1.5)
X ray	1,092 (79.9)	6,077,000	80.9 (77.6–83.9)
Any CT scan	156 (11.4)	869,000	11.6 (9.1–14.5)
Chest CT^a^	82 (8.6)	511,000	8.8 (6.1–12.5)
Drugs			
Systemic corticosteroids	893 (65.4)	4,958,000	66.0 (62.5–69.4)
Bronchodilator	955 (69.9)	5,252,000	70.0 (66.3–73.4)
Methylxanthine	4 (0.3)	20,000	0.3 (0.1–0.7)
Antibiotics	565 (41.1)	3,027,000	40.3 (36.8–44.0)
Length of ED stay, median (IQR), hr	3.5 (2.3–5.1)	3.5	2.3–5.1
ED disposition			
Admission	513 (37.6)	2,778,000	37.0 (32.4–41.8)
Hospitalization^b^			
Length of hospital stay, median (IQR), day	4.0 (3.0-6.5)	4.0	3.0–7.0
Inpatient mortality	5 (1.0)	25,000	0.9 (0.3–2.6)

^a^ from 2012–2018

^b^ Among those who were hospitalized

Abbreviations: SD = standard deviation; ED = emergency department; CT = computed tomography; IQR = interquartile range.

### ED visit rates for AECOPD

**Fig. 2:**
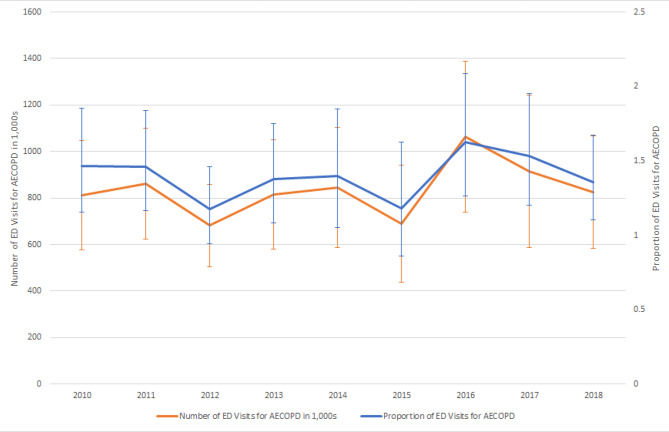
The number and proportion of emergency department visits for acute exacerbation of chronic obstructive pulmonary disease, 2010–2018

Figure 2 depicts the trend in overall ED visits for AECOPD during the study period. There were an estimated annual ED visits for AECOPD ranging from 700,000 to 1 million, and the proportions of ED visits for AECOPD were around 1.2 to 1.6% of the annual total ED visits. There was a slight increase in the proportion of ED visits for AECOPD from 2010 to 2018, but this trend was not statistically significant (P = 0.71). However, in the sensitivity analysis using more restrictive exacerbation codes, this upward trend was statistically significant (P = 0.001).

Table 2 demonstrates the ED visit rates for AECOPD stratified by patient and ED factors. The overall visit rate for AECOPD was 14 per 1,000 visits during the study period. The visit rate was lower in non-Hispanic blacks (adjusted OR [aOR], 0.6; 95%CI, 0.5–0.8) and in Hispanics (aOR, 0.4; 95%CI, 0.3–0.6). Patients with Medicare or Medicaid insurance had a higher visit rate than patients with private insurance. The visit rates were higher in the spring, fall and winter (vs. summer). The visit rate was higher in the Midwest and South (vs. the Northeast). Those who arrived in the ED by ambulance had a higher visit rate (aOR, 1.6; 95%CI, 1.4–1.9) than those who arrived by other means. The visit rates did not differ by age, sex or time of presentation to the ED.

**Table 2:** Emergency department visit rates for acute exacerbation of chronic obstructive pulmonary disease, overall, stratified, and multivariable analysis, 2010–2018

Variable	AECOPD rate per 1,000 visits	Adjusted OR (95%CI)	P value
Overall	14.0		
Age group, years			
40–64	10.7	1.0 (reference)	
65+	19.9	1.2 (0.9–1.5)	0.250
Sex			
Male	13.1	0.9 (0.8–1.1)	0.380
Female	14.7	1.0 (reference)	
Race/ethnicity			
Non-Hispanic White	16.2	1.0 (reference)	
Non-Hispanic Black	11.6	**0.6 (0.5–0.8)**	**< 0.001**
Hispanic	5.9	**0.4 (0.3–0.6)**	**< 0.001**
Other	11.3	1.0 (0.5-2.0)	0.985
Insurance			
Private insurance	7.2	1.0 (reference)	
Medicare	20.8	**2.3 (1.6–3.3)**	**< 0.001**
Medicaid or state-based program	15.0	**2.4 (1.8–3.2)**	**< 0.001**
Self-pay (uninsured)	10.0	1.4 (0.9–2.3)	0.109
Other	7.2	1.0 (0.6–1.8)	0.861
Season			
Spring (Mar. – May)	13.5	**1.3 (1.005-1.6)**	**0.045**
Summer (Jun. – Aug.)	10.7	1.0 (reference)	
Fall (Sep. – Nov.)	14.3	**1.4 (1.1–1.7)**	**0.001**
Winter (Dec. – Feb.)	17.8	**1.7 (1.4–2.2)**	**< 0.001**
Weekend			
Non-weekend	13.7	1.0 (reference)	
Weekend	14.8	1.1 (0.9–1.3)	0.297
Time of presentation			
7:00 am to 2:59 pm	14.6	1.1 (0.9–1.3)	0.266
3:00 pm to 10:59 pm	12.8	1.0 (reference)	
11:00 pm to 6:59 am	15.3	1.1 (0.9–1.4)	0.252
Geographic region			
Northeast	10.7	1.0 (reference)	
Midwest	17.9	**1.6 (1.3–2.1)**	**< 0.001**
South	15.7	**1.6 (1.2–1.9)**	**< 0.001**
West	9.4	0.9 (0.6–1.2)	0.334
Arrival mode			
Arrival not by ambulance	11.6	1.0 (reference)	
Arrival by ambulance	21.9	**1.6 (1.4–1.9)**	**< 0.001**

Significant odds ratios are highlighted in bold

Abbreviations: OR = odds ratio

*Multivariable model adjusts for all variables in the Table

### Hospitalization rates for AECOPD

**Fig. 3:**
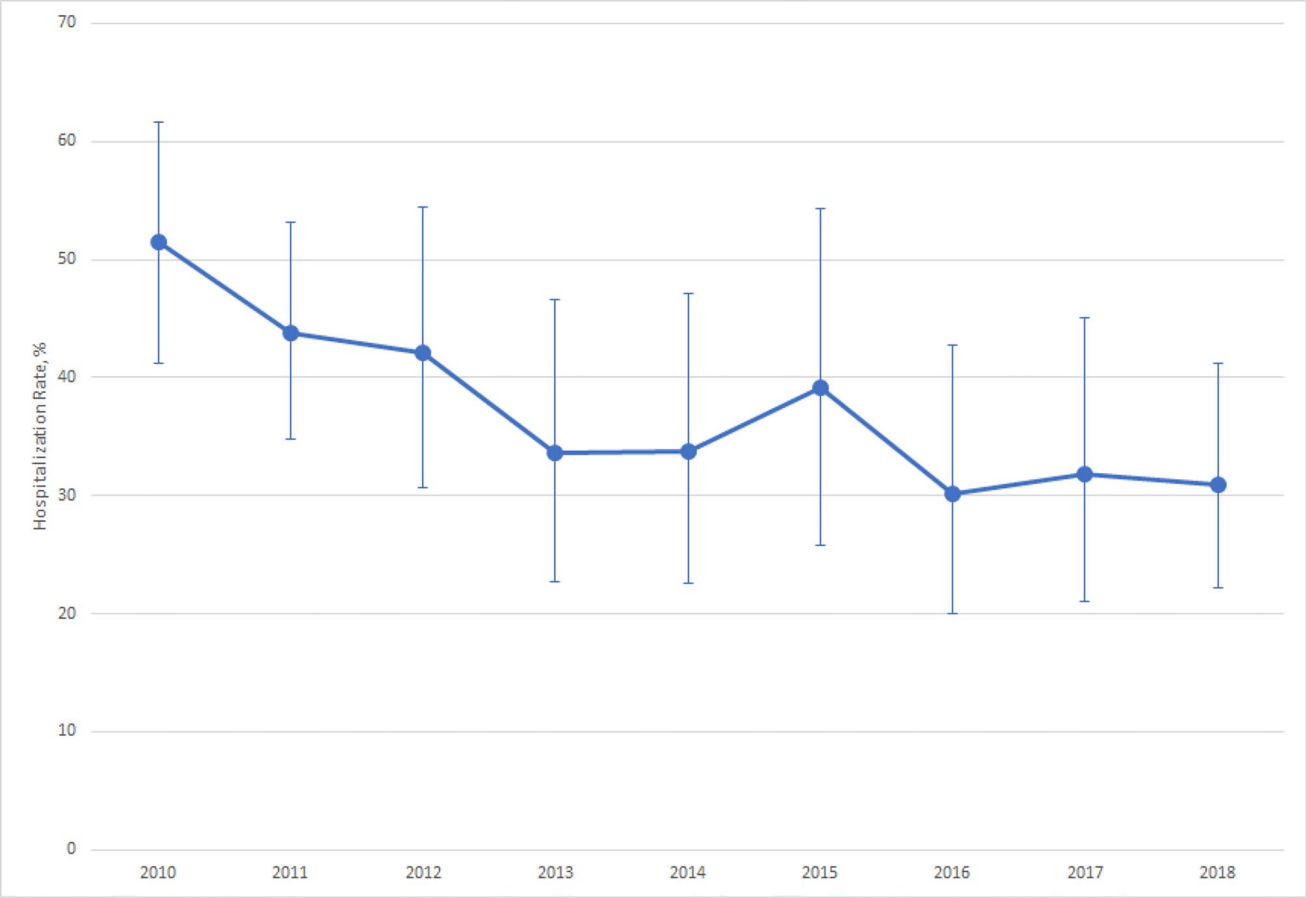
The hospitalization rate among emergency department visits for acute exacerbation of chronic obstructive pulmonary disease, 2010–2018

Figure 3 depicts the trend in hospitalization rates for AECOPD during the study period. There was a downward trend in the hospitalization rates for AECOPD from 2010 to 2018; the rate was estimated to decrease from 51% to 2010 to 31% in 2018 (P = 0.002). The decrease appeared to be linear over the years, as there was no abrupt change in the slope or intercept of hospitalization rates using regression discontinuity analysis (P = 0.18 for change in slope, P = 0.47 for change in intercept). This downward trend remained statistically significant in the sensitivity analysis (P = 0.007). Table 3 demonstrates the admission rates for AECOPD stratified by patient and ED factors. Compared with the Northeast, the admission rates were lower in the South and West (aOR, 0.4; 95%CI, 0.2–0.8 for both regions). Patients who were sent to the ED by ambulance had a higher admission rate, 53.3% (aOR, 3.2; 95%CI 2.3–4.4). The hospitalization rates did not differ by age, sex, race/ethnicity, insurance, season, or time of presentation to ED.

**Table 3:** Emergency department admission rates for acute exacerbation of chronic obstructive pulmonary disease, overall, stratified, and multivariable analysis, 2010–2018

Variable	Admission rate, %	Adjusted OR (95%CI)	P value
Overall	50.7		
Age group, years			
40–64	31.9	1.0 (reference)	
65+	41.8	1.1 (0.7–1.6)	0.652
Sex			
Male	34.1	0.8 (0.6–1.1)	0.240
Female	39.1	1.0 (reference)	
Race/ethnicity			
Non-Hispanic White	37.4	1.0 (reference)	
Non-Hispanic Black	36.6	1.2 (0.8–1.8)	0.407
Hispanic	42.5	2.0 (0.99–4.2)	0.055
Other	17.1	0.6 (0.2–1.6)	0.293
Insurance			
Private insurance	31.7	1.0 (reference)	
Medicare	42.7	1.4 (0.8–2.3)	0.242
Medicaid or state-based program	30.6	0.7 (0.4–1.4)	0.331
Self-pay (uninsured)	29.0	0.9 (0.4-2.0)	0.763
Other	32.6	1.0 (0.4–2.5)	0.980
Season			
Spring (Mar. – May)	40.0	1.2 (0.7–2.1)	0.416
Summer (Jun. – Aug.)	35.9	1.0 (reference)	
Fall (Sep. – Nov.)	34.4	1.0 (0.6–1.7)	0.860
Winter (Dec. – Feb.)	37.5	1.2 (0.7–2.2)	0.456
Weekend			
Non-weekend	38.3	1.0 (reference)	
Weekend	33.7	0.8 (0.6–1.1)	0.113
Time of presentation			
7:00 am to 2:59 pm	35.9	0.9 (0.6–1.3)	0.556
3:00 pm to 10:59 pm	39.6	1.0 (reference)	
11:00 pm to 6:59 am	34.3	0.7 (0.4–1.2)	0.163
Geographic region			
Northeast	49.2	1.0 (reference)	
Midwest	41.2	0.6 (0.3–1.2)	0.142
South	32.7	**0.4 (0.2–0.8)**	**0.005**
West	29.3	**0.4 (0.2–0.8)**	**0.009**
Arrival mode			
Arrival not by ambulance	27.7	1.0 (reference)	
Arrival by ambulance	53.3	**3.2 (2.3–4.4)**	**< 0.001**

Significant odds ratios are highlighted in bold

Abbreviations: OR = odds ratio

*Multivariable model adjusts for all variables in the Table

### Treatment with systemic corticosteroids and antibiotics for AECOPD

**Fig. 4:**
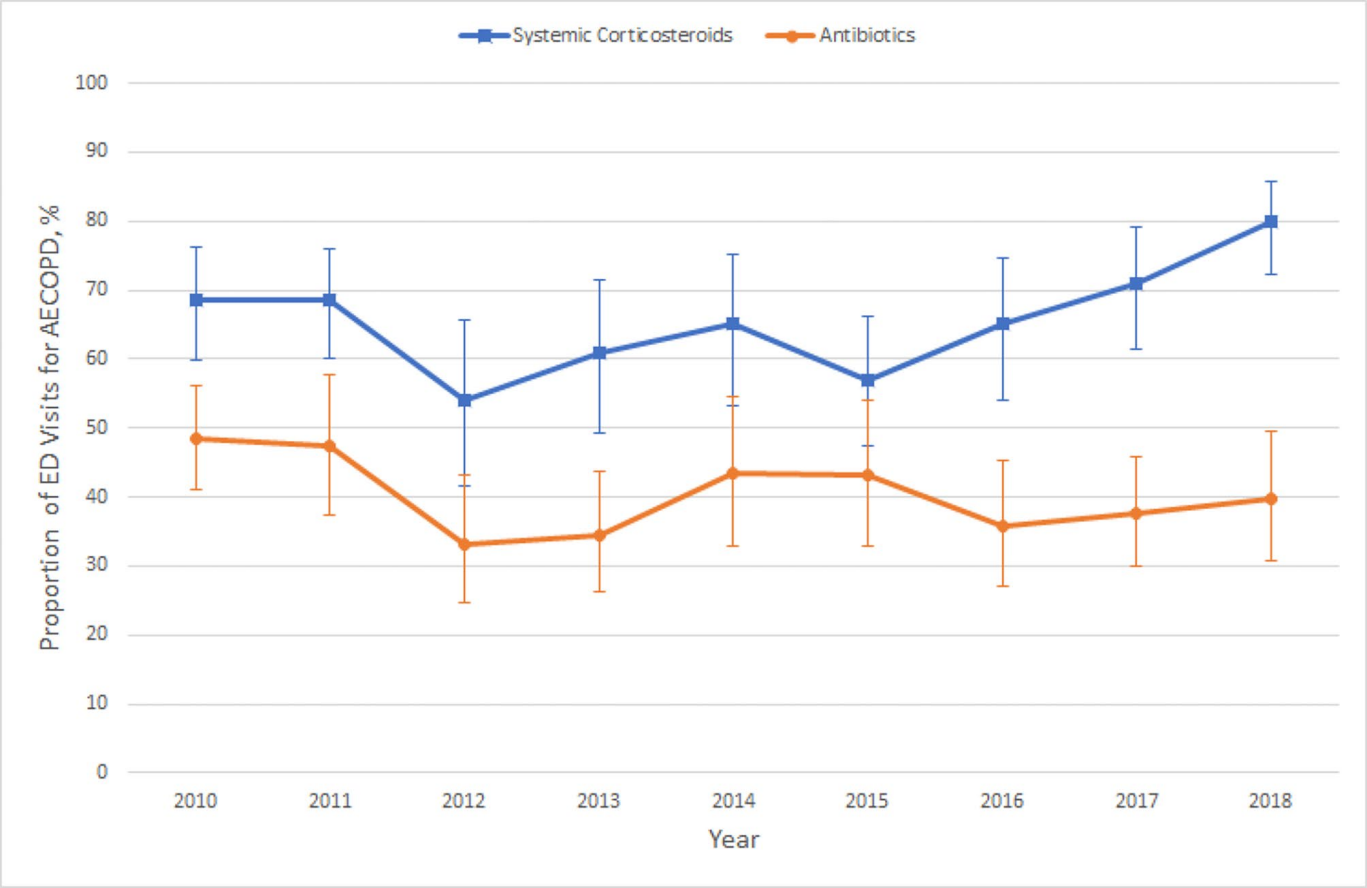
The proportion of systemic corticosteroids and antibiotics use among emergency department visits for acute exacerbation of chronic obstructive pulmonary disease, 2010–2018

Figure 4 shows the use of systemic corticosteroids and antibiotics for AECOPD in the ED from 2010 to 2018. For systemic corticosteroid treatment, there was a general upward trend during this 9-year study period, increasing to 80% in 2018 with near statistical significance (P = 0.07). For antibiotic treatment, the overall trend was relatively stable from 2010 to 2018 (P = 0.12). The results of these trend analyses remained similar in the sensitivity analysis.

## Discussion

In this national sample representative of 7,508,000 AECOPD visits, the overall ED visit rate for AECOPD remained persistently high during the 9-year study period. Certain patient and ED factors were associated with higher ED visits for AECOPD. Hospitalizations through the ED decreased over time, and there was a near statistically significant increase in the use of systemic corticosteroids in the ED during the same time period.

Compared to studies using the NHAMCS data from earlier years [4, 6], the previously identified upward trend in AECOPD ED visit rates seemed to plateau during this study period (2010–2018). A possible explanation for this trend is the combined effect of decreasing smoking rates over time in the U.S [4]., a relatively stable population of COPD [4], and perhaps improved outpatient care for patients with stable COPD. Nonetheless, there is still room for improvement. For example, the higher ED visit rate for patients with public insurance, such as Medicare and Medicaid, may suggest limitations in the current system of outpatient COPD management for this patient population and the potential benefit of targeted interventions to prevent acute COPD exacerbations [6, 18–21]. Patients with Medicaid may be more likely to experience barriers to optimal outpatient care, such as difficulty obtaining a primary care provider who accepts public insurance, challenges maintaining prescriptions for inhalers and medications, and thus rely on the ED for both unmet chronic COPD needs and management of exacerbations which are likely more frequent due to undertreated disease [6, 18–22]. Of note, interventions are especially needed during the winter months (e.g., action plan), when AECOPD rates are highest, as shown in our study and others [18, 23].

With regard to regional variations in AECOPD rates, the ED visit rates were higher in the Midwest and in the South. Similar findings were reported in an earlier study using the National Emergency Department Sample from 2009 to 2012 [10]. This regional variation may be related to differences in the prevalence of smoking and stable COPD [24], access to care (e.g., pulmonologists) [25], socioeconomic status, the severity of stable COPD, and general health status. Further study is needed to examine the associated factors and interventions (e.g., smoking cessation) to mitigate these disparities. We also found that AECOPD visits were associated with ambulance use, possibly reflecting disease severity. Similar to this study, previous research demonstrated that 65% of AECOPD patients presenting to the ED engaged in ambulance services [26]. We also noted that the ED visit rate was lower in Hispanics. This might be related to the lower prevalence of COPD in Hispanics (5%) than non-Hispanic whites (15%) [27]. In addition, the smoking rate was also lower in Hispanics (13%) than in non-Hispanic whites (17%) [28, 29].

This study showed that the hospitalization rate decreased significantly from 2010 to 2018. This result differed from an earlier study showing the total hospitalizations for AECOPD have increased from 2001 to 2012 [9], suggesting recent events that might have led to this change. The significant decrease in the admission rate might be associated with the improvement of ED management. An earlier study found that some ED management of AECOPD improved over time, particularly with the use of systemic corticosteroids and antibiotics [6]. We also extended this by reporting that the use of systemic corticosteroids continued to increase in recent years, which may, at least in part, explain the decrease in hospitalization rate. The previously identified upward trend in antibiotic use (1993–2005) seemed to plateau during this study period [6]. The stable trend in antibiotics use was consistent with a more recent (2009–2014) study reporting that less than half of the ED visits for AECOPD resulted in antibiotic therapy [30].

Interestingly, despite the fact that the ED visit rate was higher in the South, we found that the hospitalization rate in the South was actually lower. This may be due to regional differences in hospital admission practices or avoiding re-admitting AECOPD patients considering the associated financial penalties. For example, hospitals may avoid hospitalization through the ED in the first place, resulting in decreased hospitalizations. Although not statistically significant, the admissions from the ED appeared to decline before the formal inclusion of COPD as a target diagnosis in the HRRP in 2014, a finding that was consistent with a previous study using national inpatient databases [31]. This study suggested that pre-implementation “anticipatory” effects may be possible. On the other hand, however, there was a suggestion that avoiding readmissions of hospitalized AECOPD patients may cause collateral damages, such as excess mortality, particularly in safety net hospitals that care for disproportionally disadvantaged patient populations [32]. Further research is needed to clarify if there is excess mortality post-ED discharge. Lastly, we found that patients arriving in the ED by ambulance had a higher hospitalization rate. Again, this may reflect disease severity, as an Australian study has found that 78% of AECOPD patients who arrived by ambulance were admitted to the hospital, including 7% who were admitted to the intensive care unit [33].

As with all retrospective studies, this study has several limitations. First, many sources of variation can affect national estimates of ED visit rates for AECOPD, including case definitions and ICD codes. We have attempted to define the more precise patient population by using the primary diagnosis of AECOPD. Second, although the NHAMCS collected a wealth of information, some clinical granularity was lacking. Specifically, the lack of lung function test results and previous exacerbations history precluded us from detailed assessments of the stages and severity of COPD. In addition, NHAMCS data did not include the information on pre-hospital use of medications, i.e., self-medication at home or medications administered en route to the hospital (e.g., bronchodilators). This may explain the lower use of bronchodilators in our study.

## Conclusions

In summary, in this large study using 2010–2018 NHAMCS data, we provide updated information on the epidemiology of ED visits for AECOPD. The number of ED visits for AECOPD remained high; however, hospitalization for AECOPD appeared to decrease over time. Some patients were disproportionately affected by AECOPD, and certain patient and ED factors were associated with hospitalizations. Given the significant burden of disease, much work remains to be done to reduce the AECOPD ED visit rates, both at the individual level (e.g., prevention of exacerbations) and at the system level (e.g., increasing access to primary care).

Within the ED, the increasing use of systemic corticosteroids may partly explain decreased hospitalizations. Continuous quality improvement efforts in the implementation of clinical practice guidelines may further improve ED care for AECOPD. Whether decreased hospitalizations from the ED were related to the avoidance of readmissions of COPD deserves further investigation.

## Data Availability

The datasets used and/or analyzed during the current study are available from the corresponding author on reasonable request. The raw datasets can be accessed via https://www.cdc.gov/nchs/ahcd/datasets_documentation_related.htm#data
